# Case Report: FDG-PET/CT findings in co-infection of visceral leishmaniasis and chronic hepatitis B

**DOI:** 10.3389/fcimb.2023.1175897

**Published:** 2023-05-31

**Authors:** Hui Feng, Wenli Dai

**Affiliations:** Department of Nuclear Medicine, The First College of Clinical Medical Science, China Three Gorges University, Yichang, Hubei, China

**Keywords:** visceral leishmaniasis, PET/CT, hepatitis B virus infection, clinical characteristic, diagnosis

## Abstract

Visceral leishmaniasis is an opportunistic infection in immunocompromised patients. Herein, we report a case of an adult male patient with a persistent fever of unknown origin, along with chronic hepatitis B. The patient underwent bone marrow aspiration twice, which revealed hemophagocytosis. Abdomen enhanced CT revealed splenomegaly with a persistent strengthening of multiple nodules, and hemangiomas were diagnosed. A subsequent 18-fluoro-deoxyglucose (^18^F-FDG) PET/CT scan, which was implemented to search for the reason for the fever, showed diffuse splenic disease uptake, and splenic lymphoma was considered as the diagnosis. His clinical symptoms improved after receiving hemophagocytic lymphohistiocytosis (HLH) chemotherapy. However, the patient was readmitted for fever again only 2 months later. Splenectomy surgery is performed to confirm the diagnosis and classification of lymphoma. Visceral leishmaniasis was eventually diagnosed in a spleen specimen and the third bone marrow biopsy. He received treatment with lipid amphotericin B and remained recurrence-free for 1 year. In this paper, we aim to provide detailed information that will help further our understanding of the clinical symptoms and radiographic findings of visceral leishmaniasis.

## 1 Introduction

Visceral leishmaniasis (VL), also known as kala-azar, is a zoonotic parasitic disease caused by protozoan *Leishmania* parasites, mainly transmitted by the bite of female phlebotomine sandflies (Gebresilassie et al., 2015; Lévêque et al., 2020). According to the WHO, leishmaniasis is endemic in nearly 98 countries of tropical and subtropical regions, primarily affecting developing countries (Alvar et al., 2012). Since the late 1980s, western and northwestern cities of China have become the main areas where VL was endemic (Bi et al., 2018; Zhao et al., 2021). In recent years, the number of new cases of VL in central China has gradually increased (Zhao et al., 2021). The clinical manifestations of VL are usually present with prolonged irregular fever, hepatosplenomegaly, pancytopenia, and an increase in serum globulin (El Hajj et al., 2018). Moreover, VL may have led to misdiagnosis due to non-specific clinical features, worsening the condition of immunocompromised patients or even leading to death (Vechi et al., 2020). The current gold standard for VL diagnosis is based on the detection of *Leishmania* amastigotes in tissue samples (van Griensven and Diro, 2019). Currently, it is difficult to accurately diagnose VL using common imaging methods. Some patients are referred for a PET/CT scan for unknown fever. However, VL has been mostly misdiagnosed as lymphoma by nuclear medicine physicians due to the lack of systemic understanding in the past (Pinnegar et al., 2021). Thus far, only a few cases of VL complicating with other infections have been reported by PET/CT. In this case, we present the PET/CT performances of a patient with chronic hepatitis B with visceral leishmaniasis and review the relevant literature on this subject.

## 2 Case report

**Figure 1:**
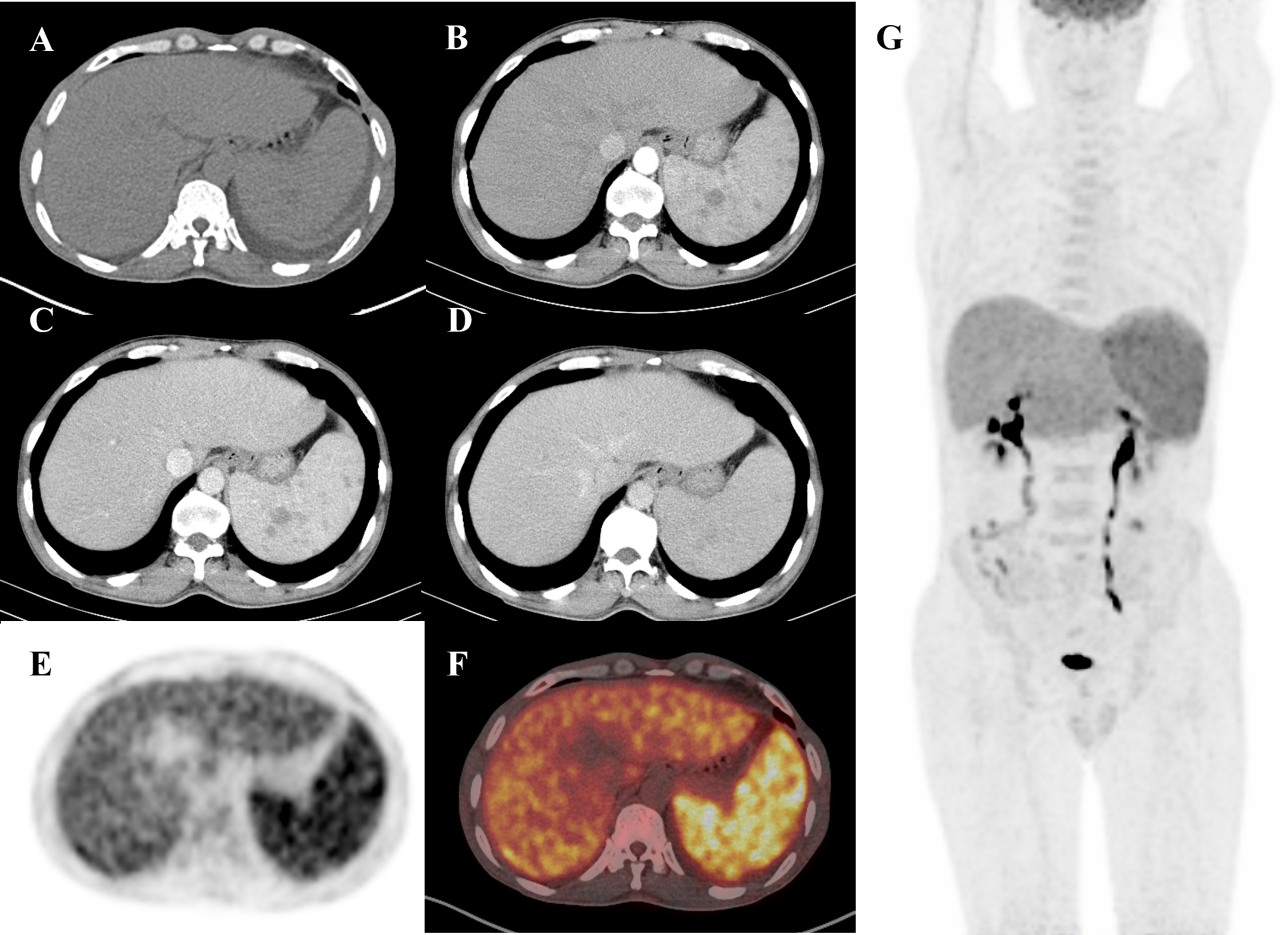
A 55-year-old man presented with a fever for 1 month. Routine diagnostic testing for fever of unknown origin (FUO) did not lead to the diagnosis of etiology. The patient was referred to the Department of Nuclear Medicine for an ^18^F-FDG PET/CT scan (axial CT, enhanced CT, PET, fusion, and maximum intensity projection (MIP)). Axial non-contrast CT **(A)** only revealed splenomegaly. However, PET **(E)** and fusion **(F)** images showed diffuse FDG uptake in the enlarged spleen with a maximum standardized uptake value (SUV max) of 6.6. The MIP image **(G)** displayed increased FDG uptake in the enlarged spleen, accompanied by multiple low-density nodules with delayed enhancement on abdominal enhanced CT **(B–D)**, which were similar to those found in lymphoma. Based on pathological examination, a diagnosis of visceral leishmaniasis (VL) was made following splenectomy. The patient received treatment with amphotericin B and was discharged from hospital without fever.

A 55-year-old man, living in Yichang City, Hubei Province, with the diagnosis of hepatitis B virus-DNA (HBV-DNA)-positive chronic severe hepatitis, was admitted to the department of hematology in the local hospital because of intermittent fever of 1-month duration; his maximum body temperature was up to 40°C. The patient had worked on a farm in Xinjiang Province a month earlier, but this clue did not raise concerns for the clinicians. The physical examination revealed hepatosplenomegaly below the costal margin. The positive findings in the laboratory results included hepatic abnormality with alanine transaminase (ALT) 490 U/L (0–40 U/L), aspartate transaminase (AST) 558 U/L (0–40 U/L), total-value bilirubin (TBIL) 23.23 μmol/L (5.1–28.0), direct bilirubin (DBIL) 14.37 (0–10), total protein (TP) 51.54 g/L (65–85), albumin (ALB) 26.83 g/L (40–55), and HBV-DNA 3.19 * 10^4^ IU/ml (<1.0 * 10^2^ IU/ml). The results of routine blood examination were slightly disordered, with white blood cell (WBC) 3.12 * 10^9^/L and platelets (PLT) 100 * 10^9^/L. An enhanced CT scan was performed, which showed an enlarged spleen with sustained strengthening multinodular (**Figures 1B–D**), and angioma was suspected. No other abnormalities in bone marrow lesions were identified by CT images. The patient received a positron emission tomography–computed tomography (PET/CT), which showed a significant diffuse increase in the uptake of FDG in the splenomegaly and slightly diffuse FDG uptake in the bone marrow (**Figures 1A, E–G**). After 2 weeks of antibiotic and cortical therapy, the patient still had intermittent fever with progressive deterioration in routine laboratory examinations including WBC 0.95 * 10^9^/L, PLT 56 * 10^9^, ALT 524 U/L (0–40 U/L), and AST 640 U/L (0–40 U/L).

In addition, bone marrow aspiration and biopsy were performed twice to confirm the previous diagnosis with an interval of 2 months, and the results showed phagocytosis of reticulum cells. Regarding these clinical and imaging findings, splenic lymphoma was highly suspected. After the chemotherapy for hemophagocytic lymphohistiocytosis (HLH), the patient was discharged from the hospital in remission. Nevertheless, he was readmitted to the hospital again for persistent fever and pancytopenia 2 months later. The patient underwent splenectomy to confirm the initial diagnosis and remove a significant burden of disease. He also received antiviral treatment with entecavir due to the history of severe hepatitis B. Subsequently, the postoperative histopathological analysis indicated diffuse proliferation of extracellular and intra-macrophage amastigotes in the spleen. At this stage, the third bone marrow biopsy was performed again, and *Leishmania* amastigotes were found. Eventually, the patient received lipid amphotericin B therapy and related symptomatic treatment, which showed excellent clinical improvement within 1 week of treatment, and his serum ALT and AST levels all dropped to the normal range. There was no recurrence of leishmaniasis over 1 year.

## 3 Discussion

VL, which is caused by protozoan *Leishmania* parasites, is mainly endemic or sporadic in Xinjiang, Gansu, Sichuan, and other regions of western and northwestern China (Bi et al., 2018). At present, the incidence of new cases of VL is increasing in central China due to warmer temperatures, deforestation or urbanization, and changes in socioeconomic factors (Oryan and Akbari, 2016; Zhao et al., 2021). Our patient lives in Hubei Province, a mid-area of China, in which VL is rarely reported before. In addition, he had worked on a farm for a month in Xinjiang Province prior to his illness, which led him to be speculated about contracting VL there.

VL is an opportunistic infection whose typical clinical manifestations involve intermittent fever, anemia, splenomegaly, and progressive leukopenia (Lachaud et al., 2009). However, immunocompromised patients with coexisting underlying conditions such as tumors, organ transplantation, immunosuppressive drug therapy, and cirrhosis may present with a variety of atypical presentations, resulting in misdiagnosis of VL and delayed treatment (Choi and Lerner, 2002; Lachaud et al., 2009). Two cases manifested as jaundice hepatitis in co-infection of VL and hepatitis B have previously been reported (Coppola et al., 2001; Godoy and Salles, 2002). Co-infected patients have more insidious clinical manifestations and longer incubation periods. One case-patient who showed VL–hepatitis B/C co-infection was readmitted to the hospital for acute jaundice hepatitis after 3 months of liposomal amphotericin treatment, and ALT and AST levels decreased after symptomatic treatment (Coppola et al., 2001). Additionally, patients with co-infection may be at risk of HBV replication and reactivation, following immunosuppression and acute hepatic necrosis when the immune response resumes (Coppola et al., 2001). Moreover, a prospective study showed significant increases in AST (>60 IU/L) and ALT (>61 IU/L), as well as total bilirubin (1.1 mg/dl), with significantly decreased levels of albumin (2.6 g/dl) and platelet count (105 * 10^3^/μl) in the co-infection of VL and hepatitis B cases. Similar to the cases described above, the serological profiles of our patients are consistent with these features, which indicated persistent liver damage (Abubakr et al., 2014). Furthermore, the treatment of VL with sodium stibogluconate (SSG) is a standardized and antimonial drug, which can cause a risk of hepatotoxicity (Mengstie et al., 2021). Thus, co-infection with VL and hepatitis B presents significant diagnosis and treatment challenges due to overlapping clinical features and hepatotoxicity of antimony-based drugs.

Imaging is less commonly used in the diagnosis of VL. To note, the enlarged spleen is a common manifestation of VL in most previously reported cases. Abdominal ultrasound and CT scan are therefore always chosen as the primary imaging modalities. The enlarged spleen lesions are most frequently hypoechoic at ultrasound images and multiple low-density nodules with persistent enhancement at contrast-enhanced CT scans (Rinaldi et al., 2019). PET/CT is valuable in the diagnosis of fever of unknown origin, but there is little information on the diagnosis potential of FDG-PET/CT in VL, especially in immunocompromised patients. PET/CT shows that most VL patients had increased spleen FDG uptake, and some patients have bone marrow and lymph node FDG uptake (Pinnegar et al., 2021). Bone marrow and lymph node uptake are more likely to occur in immunocompetent patients than in immunocompromised patients (Pinnegar et al., 2021). Two different patterns of spleen FDG uptake in VL patients have been defined on PET/CT examination: diffuse and focal (Zanoni et al., 2019). These features need to be differentiated from other similar diseases of splenic FDG uptake, such as splenic marginal zone lymphoma and infectious granulomatous disease. In this case, our patient subsequently underwent splenectomy to identify the etiology and alleviate symptoms. Finally, postoperative pathology confirms the diagnosis of VL. Removal of a large number of parasites is one of the potential benefits of splenectomy, as the level of drugs used to treat VL in the enlarged spleen may not be sufficient to eradicate the parasite and cure the disease in the presence of severe splenomegaly. Splenectomy may be considered for curative purposes in patients with clinical impact on hypersplenism and coagulopathy (Troya et al., 2007; Reinaldo et al., 2022). Therefore, while PET/CT may be useful for early intervention in suspected VL cases, its value in guiding splenectomy needs further study. We stress the need for comprehensive assessment and careful decision-making in cases where diagnosis and treatment options are ambiguous.

Furthermore, VL has a longer incubation period in mostly HIV-positive and solid organ transplant patients, which even shows *Leishmania* detected by atypical locations other than the spleen, liver, or bone marrow (Pinnegar et al., 2021). It was previously reported that an immunosuppressed patient presented an incubation period of VL over 15 years, and his PET/CT showed enhanced uptake throughout the colon, spleen, liver, lymph nodes, and lungs (Rinaldi et al., 2019). Such imaging findings might occur only when the parasitic load is high due to long-standing infection and prolonged immunosuppressive therapy. Although our patient had co-infection of VL and hepatitis B, PET/CT only manifested as diffuse uptake of an enlarged spleen, which was consistent with most previously reported cases. Remarkably, retrospectively evaluating three bone marrow pathologic results, we observed that amastigotes were only detected in bone marrow biopsy after splenectomy.

When female sandflies interact with the host skin and inject promastigotes into the dermis, the infection begins. During the interaction between the parasite and host macrophages, *Leishmania* promastigotes can evade or disrupt host immune responses by inducing inflammatory mediators, interfering with signal transduction pathways, and achieving survival and persistence (Bogdan and Röllinghoff, 1998; Conceição-Silva and Morgado, 2019). Once the parasite transforms into intracellular amastigotes, the next step is transmission. Asymptomatic amastigote infection can lead to increased parasitic load (Wenzel et al., 2012). Pathogenic amastigotes proliferate in macrophages, causing massive destruction and proliferation of macrophages, mainly in the spleen, liver, lymph nodes, and bone marrow. Splenomegaly, caused by cell proliferation, is the most common. The findings of Beattie et al. suggested that under certain inflammatory conditions, such as obsessive liver inflammation, bone marrow (BM)-derived monocytes can differentiate into tissue-resident macrophages, such as KC, and contribute to the host immune responses (Beattie et al., 2016). The process of recruitment and differentiation of BM-derived monocytes is critical for the regression of infection and tissue repair after injury (Zigmond et al., 2014). In the case of *Leishmania* infection, it is known that both yolk sac (YS)-derived and BM-derived macrophages are involved in the immune response. Several studies have shown the role of YS-derived macrophages in the early control of infection and the role of BM-derived macrophages in late infection and disease resolution (Protzer et al., 2012; Yona et al., 2013). Therefore, based on the above research background, we hypothesize that splenectomy not only reduces the parasite burden in co-infection patients of VL and hepatitis B but also results in the loss of a large number of macrophages that respond to parasitic infections. However, the parasites in the host’s body are not completely eliminated. *Leishmania* amastigotes continue to replicate and induce inflammation, further triggering BM-derived monocytes to proliferate and convert to KC in response to parasitic infection. This may indirectly reflect the results of our patient’s third bone marrow biopsy, which showed the absence of amastigotes. Overall, BM-derived monocytes recruit and differentiate into tissue-resident macrophages that play an important role in the immune response to infection and post-injury tissue repair. The ability of BM-derived monocytes to differentiate into resident tissue macrophages such as KCs in the context of *Leishmania* infection has not been extensively studied, but this is an area of interest for future research.

Although examining tissue smears under the microscope is a cost-effective, minimally invasive method widely used in primary healthcare systems, it can only provide partial quantitative information on parasite load and cannot distinguish *Leishmania* species (da Silva et al., 2005). In addition, the sensitivity of this method is highly dependent on the number and dispersion of parasites in biopsy tissue. In addition, Barros Pinto et al. suggested that the transformation from amastigotes to promastigotes that occurs *in vitro* may be related to a decrease in temperature and pH changes in bone marrow samples. They recommend that bone marrow smears be performed as soon as possible after collection to avoid delayed diagnosis due to morphological changes in *Leishmania* parasites *in vitro* (Barros Pinto et al., 2021).

In summary, VL coexisting with other underlying conditions may complicate the diagnosis, especially in immunocompromised patients. It is necessary to be cautious in identifying VL in clinical practice. VL should be included in the differential diagnosis when patients have a history of travel to endemic areas, irregular fever, concomitant immunocompromised disease, and increased spleen FDG uptake on PET/CT.

## Data availability statement

The original contributions presented in the study are included in the article. Further inquiries can be directed to the respective author.

## Ethics statement

Ethical review and approval was not required for the study on human participants in accordance with the local legislation and institutional requirements. The patients/participants provided their written informed consent to participate in this study. Written informed consent was obtained from the individual(s) for the publication of any potentially identifiable images or data included in this article.

## Author contributions

HF dealt with the case and drafted the manuscript. WD participated in the revision of the manuscript for important intellectual content. All authors contributed to the article and approved the submitted version.
